# Validation of the adapted pregnancy-related anxiety scale in Northern Ghana (PrAS-NG)

**DOI:** 10.18332/ejm/215188

**Published:** 2026-03-20

**Authors:** Majd K. Qannita, Gilbert Abotisem Abiiro, Dominic Akaateba, Kelly Hadfield, Kristin Hadfield

**Affiliations:** 1 School of Psychology, Trinity Centre for Global Health, Trinity College Dublin, Dublin, Ireland; 2 Department of Health Services, Policy, Planning, Management and Economics, School of Public Health, University for Development Studies, Tamale, Ghana; 3 Ghana Medical Help, Accra, Ghana

**Keywords:** reliability, cross-cultural validation, measurement invariance, pregnancy-related anxiety scale, Northern Ghana

## Abstract

**INTRODUCTION:**

Pregnancy-related anxiety refers to anxiety experienced throughout pregnancy, including concerns about labor, maternal and fetal wellbeing, healthcare access, and parental readiness. Pregnancy-related anxiety has significant adverse effects on maternal and neonatal outcomes, distinct from other mental health conditions during pregnancy. However, research in low- and middle-income countries, notably Ghana, remains limited, in part because of lack of culturally relevant, high-quality measures. This study aims to validate the adapted PrAS for use in Northern Ghana (PrAS-NG).

**METHODS:**

Using survey data from 586 pregnant women, we conducted a pre-registered assessment of the reliability and validity of the PrAS-NG. Reliability was evaluated using Cronbach's alpha and test-retest analyses. For internal validity, we conduct exploratory factor analysis and confirmatory factor analysis. Convergent and divergent validity were assessed through Pearson correlation analysis.

**RESULTS:**

Following an exploratory factor analysis of the initial 52-item scale, a refined, 22-item, 9-factor version (PrAS-NG22) was developed. A confirmatory factor analysis validated the scale with a comparative fit index of 0.96. The scale demonstrated full invariance across age, gestational age, and parity. Internal (α=0.90) and test-retest reliability were strong (ICC=0.81). The PrAS-NG22 was related to anxiety and psychological distress during pregnancy, suggestive of convergent validity.

**CONCLUSIONS:**

The results suggest the PrAS-NG22 is a valid, reliable, and culturally sensitive tool for pregnancy-related anxiety screening in Northern Ghana.

## INTRODUCTION

Pregnancy is a highly susceptible period for mental health issues due to physiological and psychological changes^1^. Pregnancy-related Anxiety (PrA) refers to excessive worry and fear concerning the baby’s health, the mother’s health and physical changes, interactions with the healthcare system, as well as social and financial challenges related to pregnancy, childbirth, and parenting^2^. PrA can significantly impact maternal health, influencing pregnancy, childbirth, and the wellbeing of both mothers and newborns^3^. Specifically, PrA is linked to increased risks of maternal mortality, preterm labor, low birth weight, difficulties with breastfeeding, postpartum depression, anxiety, poor maternal-child bonding, and developmental impairments in children^4^. PrA is uniquely associated with these adverse maternal and neonatal outcomes, over and above the effects on antenatal depression or anxiety^3^. Pregnant women with PrA face specific concerns that require focused attention^2^. Globally, anxiety disorders are present in 15% of pregnant women, with the highest occurrence during the third trimester^5^. In the meta-analysis of Dennis et al.^5^, women in low- and middle-income countries (LMICs) had a higher incidence of anxiety disorders during pregnancy than those in high-income countries (HICs), with rates of 34.9% and 19.8%, respectively. The differential prevalence between LMICs and HICs was explained by factors such as education level, economic situation, health system characteristics, household power dynamics, and cultural norms^6^. However, research on pregnancy-related anxiety in LMICs is limited, and may be hampered by ineffective or non-culturally relevant measurement^7^. The concerns of pregnant women in LMICs, such as maternal mortality and healthcare access, differ from those in HICs, where the focus is on body image, miscarriage, and parenting^2,8^. Therefore, tools developed for HICs may not effectively capture the unique aspects of PrA in LMICs^9^. A review by Hadfield et al.^10^ identified the Pregnancy-related Anxiety Questionnaire, Pregnancy-related Anxiety Scale, and Cambridge Worry Scale as the most common tools for PrA. However, assessment is mostly limited to HICs, with locally developed or validated measures lacking in LMICs^11^. Indeed, only two measures were developed in any LMIC, and only four were validated for use in these settings – none of these was developed or validated in Africa^10^. This highlights the need for culturally sensitive, psychometrically validated tools to assess PrA in Africa.

The Pregnancy-related Anxiety Scale (PrAS) was developed to assess pregnancy-related anxiety in Australia^12^. The scale includes 33 items across eight factors: childbirth concerns, body image concerns, attitudes towards childbirth, worry about motherhood, acceptance of pregnancy, anxiety indicators, attitudes towards medical staff, and baby concerns. This scale introduced new areas not addressed in previous tools. Psychometric evaluations showed good content validity and strong internal consistency, with reliability ranging from good to excellent. Brunton et al.^12^ emphasized the need for cultural adaptation of the PrAS, which has been applied to populations like Turkish and Japanese pregnant women^12,13^.

In Ghana, there is a need for valid and reliable tools to measure PrA. In response, a project was launched in 2021 to adapt the PrAS to the cultural context of Northern Ghana, using local languages (Dagbani and English) and culturally salient items. In this manuscript, we describe our methods for psychometrically validating this measure: evaluating the validity, reliability, and measurement invariance of PrAS-NG in the Northern Ghana context.

## METHODS

### Ethics considerations

Ethical approval for the original data collection was granted by the Ghana Health Service Ethics Review Committee (GHS-ERC 010/02/21), with additional permission from the Northern Regional Health Directorate and Tamale Teaching Hospital. Informed consent was obtained from all participants. The current study uses data which were accessed on the Open Science Framework (OSF) (https://osf.io/rd56g/?view_only=83a0ba3a6bee41b48bb0055729283073).

### Design

**Figure 1:**
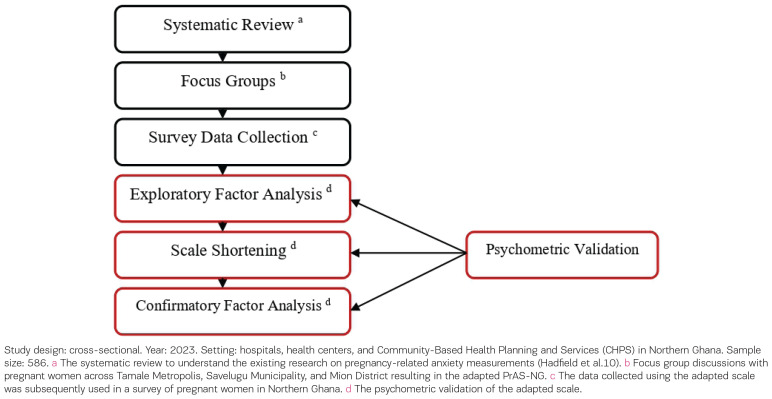
Stages in the development of the Pregnancy-related Anxiety Scale (PrAS) in Northern Ghana

This study is a cross-sectional validation analyzing survey data collected as part of a study that adapted the PrAS items (Figure 1) among pregnant women attending antenatal clinics in the Tamale Metropol, Savelugu Municipal, and Mion districts, in Northern Ghana. We collected qualitative data and analyzed this to develop the adapted PrAS (described below and in Abiiro et al.^14^) and this adapted survey was then implemented with a sample of pregnant women.

### Qualitative work

The original 32-item PrAS was translated into Dagbani, then back-translated into English, and versions were compared by the research team and a local advisory committee. As part of this earlier qualitative component, 15 focus group discussions with pregnant women were conducted in the Tamale Metropol, Savelugu Municipal, and Mion districts to assess content validity, clarity, and cultural relevance of the PrAS. There were 108 participants in the focus groups conducted in the earlier adaptation study (age range 16–39 years; mean=26, SD=5.1). Women were recruited from Tamale Metropol (n=18), Savelugu District (n=47), and Mion District (n=43); 67.9% lived in rural areas and 42.3% were in a polygamous marriage.

Thematic analysis of this focus group data led to removal of eight items and the addition of 27 items that reflected local concerns about pregnancy, health staff attitudes, spirituality, partner and household support, stigma, financial burden, transportation, and naming ceremonies. This process produced a 52-item draft PrAS-NG covering 14 domains. These qualitative data are not analyzed in the present study, but are summarized here to describe the development of the adapted instrument; further details are reported in Abiiro et al.^13^. We then translated the entire 52-item scale (PrAS-NG) to be in English and Dagbani using a team translation framework to maintain conceptual equivalence between languages, and then implemented it in a survey sample.

### Scale validation

The adapted 52-item scale was administered between November and December 2021 in a cross-sectional survey using face-to-face interviews and Kobo Toolbox, in English or Dagbani according to participant preference (92.7% chose Dagbani). A subsample of 45 women were re-contacted for a retest interview by phone, stratified by district and facility type. These survey data provide the dataset for the current validation analyses.

### Participants

The survey sample used for the current cross-sectional validation comprised 586 pregnant women from the same three districts: Savelugu (40.8%, n=239), Mion (30.7%, n=180), and Tamale Metropol (28.5%, n=167). Most participants were married (96.2%, n=563), lived in rural areas (64.5%, n=377), and had no formal education (77.8%, n=456). Overall, the survey participants represent a typical group of women receiving antenatal care in these districts.

### Measures and variables

*Demographics*

A participant’s age was determined by asking: ‘How old were you on your previous birthday?’. If unclear, age was verified through the Maternal and Child Health Record Book (MCHRB) or insurance card. Gestational age was obtained by the response to the question: ‘How many months into your pregnancy are you?’, if the respondent was unsure, it was extracted from the MCHRB, where possible. Clinically, the gestational age is defined into three trimesters. The first trimester is weeks 1–12 (or months 1–3), the second trimester is weeks 13–27 (or months 4–6), and the third trimester is weeks 28–40 (or months 7–9)^15^. Participants were asked if this was their first pregnancy and were divided into two groups: nulliparous women (those with no previous pregnancies lasting >20 weeks) and parous women (those with prior pregnancies, whether primiparous or multiparous)^15^.

*Mental Health*

Generalized anxiety

The 2-item Generalized Anxiety Disorder Scale (GAD-2) was used to assess anxiety symptoms^16^. GAD-2 is valid for African women^17^. The GAD-2 includes the first two items from the 7-item GAD-7^18^ assessing frequency of ‘feeling nervous, anxious, or on edge’ and ‘being unable to stop worrying’ over the past two weeks. Responses are rated on a Likert scale from 1 (not at all) to 4 (nearly every day). The total score ranges from 2 to 8, with higher scores indicating greater anxiety. Internal reliability in this sample was α=0.72.

Wellbeing

Mental wellbeing was assessed using the 7-item short version of the Warwick Edinburgh Mental Wellbeing Scale (WEMWBS). WEMWBS was validated among the African population^19^. The scale includes items such as: ‘I have been feeling optimistic about the future’ and ‘I have been feeling relaxed’. Participants rated their agreement with each item on a Likert scale from 1 (none of the time) to 5 (all of the time). Sum scores from 7 to 35, with higher scores indicating greater wellbeing. Internal reliability in this sample was α=0.82.

Depression

Depressive symptoms were assessed using the Edinburgh Postnatal Depression Scale (EPDS), a valid widely deployed self-report tool for assessing depression during pregnancy^20^. The scale consists of ten items, including statements such as: ‘I have blamed myself unnecessarily when things went wrong’ and ‘I have felt sad or miserable’. Participants rated the severity of each item over the past week on a four-point Likert scale (1 to 4), with lower scores indicating more depressive symptoms. The EPDS demonstrated good reliability in this sample (α=0.81).

Psychological distress

Psychological distress was assessed using the Kessler 6-item Psychological Distress Scale (Kessler-6), commonly used in studies on distress during pregnancy, especially in Africa^20^. Participants rated distress symptoms experienced in the past 30 days on a scale from 1 (all of the time) to 5 (none of the time) for six items: nervousness, worthlessness, restlessness, depression, effort, and feeling worthless. Scores ranged from 5 to 25, with lower scores indicating greater distress. The internal reliability of the measure was good (α=0.81).

Pregnancy-related anxiety

Pregnancy-related anxiety was assessed using the PrAS-NG scale, which originally consisted of 52 items (Supplementary file Appendix A). Participants rated each item on a scale from 1 (not at all) to 4 (very often), with scores ranging from 52 to 208. Higher scores indicated greater anxiety. Following exploratory factor analysis (EFA), the scale was reduced to 22 items, with both English and Dagbani versions provided in Table 1.

**Table 1:** The shortened Pregnancy-related Anxiety Scale–NG22 (PrAS-NG22) in English and Dagbani used among pregnant women in Northern Ghana

*Items*	*English*	*Dagbani*
	**Factor 1. Birth preparedness**	
**PrAS1**	I worry that I won’t be able to organize clothes for my baby before my delivery date.	Di teharimami ni nkutoi laɣisi m-bileɣu maa nema pɔin ka o sheena.
**PrAS2**	I worry about not getting all the necessary delivery items before my delivery date.	Di teharimami ni nkutoi laɣim doɣim bin chehi chehi pɔin ka ndoɣim saha paagi.
	**Factor 2. Medical physical pain**	
**PrAS3**	I worry about unnecessary interventions during delivery (e.g. forceps use during delivery).	Di teharimami zanῄkpa vihigu taligu nitiyen dɔɣi (eg maɣsi yuusibu).
**PrAS4**	I worry that I will tear or need to be cut during child birth.	Di teharimami ni n-tooni nitoi tahi bei kabi pahi ntooni niti yen dɔɣi.
	**Factor 3. Delivery concern**	
**PrAS5**	I worry that the midwives will slap/hit my thighs while in labor.	Di teharimami ni nursi nimmaa (midwives) ni ῄmema gbinpaɣasi bei ῄme n-gbalipina tapaɣsi n doɣim saha.
**PrAS6**	I worry that I will not be able to deliver per vagina.	Di teharimami ni nkutoi doɣi tooni doɣibu.
**PrAS7**	I worry that I will have miscarriage.	Di teharimami ni ni ntoi zanῄ puliῄo ndoli shinsheɣu.
	**Factor 4. Health workers attitudes**	
**PrAS8**	I know that midwives/doctors will be kind.	Mmi ni doctaninmaa ni nursenimaa bori niriba yeltoɣa.
**PrAS9**	I know that midwives/doctors will be helpful.	Mmi ni doctaninmaa ni nursenimaa ni sonῄma.
**PrAS10**	I know that I can ask the midwives/doctors anything.	Mmi ni nitoi bohi doctaninmaa ni nurseninmaa binsheɣu kam.
	**Factor 5. Psychological/social pressure**	
**PrAS11**	I worry that I will be stigmatized if I have to go through CS.	Di teharimami ni bin ganῄbu mandei ni dɔɣi operation dɔɣibu.
**PrAS12**	I worry that people will think that I am weak if I deliver in the hospital.	Di teharimami ni niribi ni tehi ni anka yaa nninῄgbinani mandei ni dɔɣi ashiptini.
**PrAS13**	I worry that my husband may think that I am unfaithful if I am unable to deliver per vagina.	Di teharimami ni nyidan nitoi tehari ni nyila sambani dindeeni ninῄ kanbitoi doɣi tooni dɔɣim.
	**Factor 6. Resources availability**	
**PrAS14**	I worry about the cost of transport to the health facility for ante-natal care or delivery.	Di teharimami zankpa laɣifu sheli ni yen di nchang zahimbu bei n-kpuɣi loori bei motor nchang zahimbu bei ni yen doɣi.
**PrAS15**	I worry that I won’t get access to a vehicle if I am referred to a higher facility to deliver.	Di teharimami ni nku-nya loori nchang ashipti titalini ashipti bila nim ni yeli ni nchamg ashipti titalini.
**PrAS16**	I fear that I won’t get enough support from my husband or partner during my pregnancy.	Dabem malimami ni nyidana ku sonῄma venyela npulli ῄo shei.
	**Factor 7. Baby concerns**	
**PrAS17**	I worry about what I will do if my baby is not normal.	Di teharimami niyen n-ninῄ shem dindeening ka n-bii maa nahim gbana bi paaigi.
**PrAS18**	I worry about having a sick or disabled baby.	Di teharimami ni n-bii maa nni tooi ka alaafei bei ka o mali dalinῄ.
	**Factor 8. Financial concerns**	
**PrAS19**	I worry about the cost of childbirth.	Di teharimami zanῄkpa dɔɣim laɣi chehi chei dibu.
**PrAS20**	I worry that I may have to pay out of pocket for childbirth.	Di teharimami ni ndɔɣim saha ashipti nimmaa ni cheka nyo laɣisheῄa.
	**Factor 9. Maternal and neonatal mortality concerns**	
**PrAS21**	I worry that I may lose my life through childbirth.	Di teharimami ni ntoi kani dɔɣim saha.
**PrAS22**	I worry that I will have still birth.	Di teharimami ni ni ntoi dɔɣi mbii maa ka oka nyevili.

Study design: cross-sectional. Year: 2025. Setting: hospitals, health centers, and Community-Based Health Planning and Services (CHPS) in Northern Ghana. Sample size: 586.

### Statistical analysis

Analyses were conducted using SPSS v.28 and MPlus v.8.1. The construct validity of the PrAS-NG was assessed by EFA, confirmatory factor analysis (CFA), and discriminant and convergent validity.

*Validity*

We randomly split the sample into two groups of 293 participants and then conducted an EFA to determine the factor structure of the scale. Maximum likelihood with robust standard errors (MLR) estimation was used due to its robustness for sample size and non-normality^21^. We used the following criteria to determine the best model: Eigenvalue >1; non-significant χ^2^; χ^2^:df ratio <3:1; CFI and TLI ≥0.90 (acceptable) and ≥0.95 (good); RMSEA ≤0.08 (acceptable) and ≤0.06 (good); SRMR ≤0.10 (acceptable) and ≤0.08 (good)^22^.

AIC and BIC were also used to compare models, with the smallest value indicating the best fit. The initial EFA factor structure included 27 items. Items with factor loadings more than 0.4 were retained. A reduction process selected 2–3 items per factor, prioritizing those with higher loadings to ensure the scale accurately represented the construct. The final version, PrAS-NG22, comprised 22 items and nine factors. A CFA was conducted in the second random half of the sample, evaluating the validity of the PrAS-NG22 as identified in the EFA. Multivariate normality was assessed using Mahalanobis distance, with outliers evaluated against the χ^2^ distribution. The CFA assessed using the same cutoffs in the EFA model. Finally, a multi-group CFA was conducted to assess measurement invariance, which evaluates whether the tool measures the construct consistently across subgroups^21^. Invariance was tested for configural, metric, and scalar levels. Configural invariance ensures a similar factor structure across groups, metric invariance indicates equivalent factor loadings, and scalar invariance means the baseline for the scale is the same, allowing for meaningful comparison of average scores across groups^21^. In this series of multi-group CFAs, invariance was assessed across age (≤24 vs >24 years), trimester (first/second vs third), and parity status (nulliparous vs primiparous/multiparous) groups. A separate CFA was conducted for each group to compare model fit with the initial population-level CFA, assessing configural invariance. Chi-squared difference tests compared metric, scalar, and configural invariance. The configural model served as the baseline with no constraints, while the metric model constrained factor loadings, and the scalar model constrained both loadings and intercepts. Model fit indices (CFI, TLI, RMSEA) were assessed for each model. CFA mean differences and Wald tests were conducted for factor correlations. Full invariance was considered if the following criteria were met: CFI and TLI differences <0.01, non-significant chi-squared differences between models, RMSEA differences ≤0.015, and non-significant Wald test results^22^.

Finally, convergent and discriminant validity were assessed in the full sample (n=586) using Pearson correlations. Convergent validity was evaluated by examining the correlations between PrAS-NG22, GAD-2, EPDS, and Kessler-6. Discriminant validity was assessed by checking for a significant negative correlation between the PrAS-NG22 and the WEMWBS.

*Reliability*

The reliability of the PrAS-NG was assessed through internal and test-retest reliability. Test-retest reliability, assessing stability over time, was evaluated by administering the PrAS-NG to 45 pregnant women on two separate occasions, separated by approximately a week. The interclass correlation coefficient (ICC) was calculated using SPSS, with values between 0.7 and 0.9 indicating acceptable test-retest reliability^23^.

## RESULTS

### Construct validity

*Exploratory factor analysis*

After reverse coding to the positively worded items, which are (PrAS3, PrAS6, PrAS7, PrAS18, PrAS19, PrAS21, PrAS22, PrAS23, PrAS24, PrAS25, PrAS26, PrAS40), 12 factors were extracted based on eigenvalues <1. The 9-factor model exhibited the most favorable fit indices among all the factor models tested. To illustrate, CFI=0.906, TLI=0.861, RMSEA=0.047, the SRMR=0.034, χ^2^: df=1.62:1, AIC=37332.529, and BIC=39305.101 indicating a good fit with the data and a less complex representation of the data than alternative models.

After examining the oblique rotated factor loadings, the following indicators (items) were excluded from the analysis: PrAS 3, PrAS 6, PrAS 7, PrAS 8, PrAS 9, PrAS 10, PrAS 11, PrAS 12, PrAS 14, PrAS 16, PrAS 17, PrAS 18, PrAS 19, PrAS 20, PrAS21, PrAS25, PrAS 26, PrAS27, PrAS 33, PrAS 36, PRAS 40, PRAS 41, PRAS 42, PRAS 43, PRAS 44, PRAS 46, PrAS49, PrAS50, PRAS 51, and PRAS 52. The shorter version (PrAS-NG22) of the tool represents nine distinct factors and 22 items (Table 1).

*Confirmatory factor analysis*

Only two cases in the CFA subsample (n=293) exceeded the Mahalanobis distance threshold [χ^2^(22)=48.27, p<0.001], representing <1% of the sample and suggesting no meaningful violation of multivariate normality. The CFA for the PrAS-NG22 showed good model fit: χ^2^(276)=3192.06, p<0.001; CFI=0.957; TLI=0.943; RMSEA=0.045; and SRMR=0.050. For comparison, the original PrAS-NG demonstrated: χ^2^(351)=3523.10, p<0.001; CFI=0.940; TLI=0.927; RMSEA=0.047; and SRMR=0.057. Accordingly, thePrAS-NG22 demonstrated improved overall fit based on higher CFI and TLI values and lower RMSEA and SRMR. See Supplementary file Appendix B for the final factor model for the PrAS-NG22.

### Measurement invariance

*Measurement invariance by age groups*

Because women aged <24 years have an elevated risk of experiencing PrA^24^, we categorized participants from the CFA sample by age (≤24 years, younger group, n=96; >24 years, older group, n=196). In the context of multi-group CFA, it is recommended that each sub-group have a minimum threshold of 100 observations^25^. The fit indices for the younger age group CFI=0.914, RMSEA=0.067 and TLI=0.921, and for the older age group, CFI=0.952, RMSEA=0.048, and TLI=0.941indicated an acceptable fit. However, the CFI, RMSEA, and TLI differences exceeded the threshold, mostly due to small samples. Invariance testing showed no significant differences between the configural, metric, and scalar models (p>0.05), which suggests full measurement invariance across age groups (Table 2). The older group had a significantly higher mean score in the health workers attitudes’ factor (estimates=0.376; p=0.000). Furthermore, the Wald test yielded non-significant results (value=0.026, df=1, p=0.8730). These findings collectively indicated that the PrAS-NG22 demonstrated full invariance across different age groups.

**Table 2:** Measurement invariance of the adapted Pregnancy-related Anxiety Scale (PrAS-G22) across two age groups among pregnant women in Northern Ghana

	*χ^2^*	*df*	*CFI*	*TLI*	*RMSEA*	*ΔCFI*	*ΔTLI*	*ΔRMSEA*	
**Configural**	498.583*	346	0.939	0.919	0.055	**0.005**	0.011	**0.004**	1 vs 3
**Metric**	501.778*	359	0.943	0.927	0.052	**0.004**	**0.008**	**0.003**	2 vs 1
**Scalar**	513.390*	372	0.944	0.930	0.051	**0.001**	**0.003**	**0.001**	3 vs 2

Bold values indicate that invariance was met. Younger women: ≤24 years (n=96); older women: >24 years (n=196). Study design: cross-sectional. Year: 2025. Setting: hospitals, health centers, and Community-Based Health Planning and Services (CHPS) in Northern Ghana. Sample size: 293.

* p<0.05.

*Measurement invariance by parity groups*

The sample comprised 60 primiparous and 233 multiparous women. The fit indices for the combined primiparous/multiparous group were CFI=0.944, RMSEA=0.051, and TLI=0.901, better than the nulliparous group where CFI=0.892, RMSEA=0.082, and TLI=0.886. The high CFI, TLI and RMSEA difference (0.08, 0.015,0.031, respectively) could be correlated to the smaller sample size of the nulliparous group. There was no significant difference between the configural, scalar, and metric models (p>0.05) (Table 3). The nulliparous group had lower mean scores in the health workers’ attitudes factor (estimates= -0.239, p=0.029) and in the resources availability factor (estimates= -0.325, p=0.010). Finally, the Wald test showed non-significant results (value=0.046, df=1, p=0.8303). This suggests full measurement invariance for nulliparous versus primiparous or multiparous women, in the PrAS-NG22.

**Table 3:** Measurement invariance of the shortened Pregnancy-related Anxiety Scale (PrAS-NG22) across parity groups of pregnant women in Northern Ghana

	*χ^2^*	*df*	*CFI*	*TLI*	*RMSEA*	*ΔCFI*	*ΔTLI*	*ΔRMSEA*	
**Configural**	523.128*	346	0.931	0.908	0.059	0.003	0.01	0.01	1 vs 3
**Metric**	526.772*	359	0.935	0.916	0.056	0.004	0.008	0.003	2 vs 1
**Scalar**	542.448*	372	0.934	0.918	0.069	0.001	0.002	0.013	3 vs 2

Bold values indicate full invariance was met. Study design: cross-sectional. Year: 2025. Setting: hospitals, health centers, and Community-Based Health Planning and Services (CHPS) in Northern Ghana. Sample size: 293. The sample included nulliparous women (n=60) and primiparous/multiparous women (n=233).

* p<0.05.

*Measurement invariance by gestation*

We split the sample into third trimester and first/second trimesters based on literature indicating higher PrA rates in the third and the recommended CFA sample size for multiple groups^25,26^. There were 139 participants in the first and second trimesters and 154 participants in the third. For the first/second trimester group CFI=0.925, TLI=0.913 and RMSEA= 0.063, whereas for the third-trimester group CFI=0.940, TLI=0.932 and RMSEA=0.054. The 0.015 CFI and 0.018 TLI differences slightly exceed the threshold, but the 0.009 RMSEA difference suggests configural invariance. The configural, metric, and scalar comparisons were not significant (p>0.05) except for scalar against metric (p=0.0046) (Table 4). However, the differences between CFIs and RMSEA were less than 0.01 and 0.015, respectively. Accordingly, full invariance was demonstrated. The third-trimester group showed non-significant mean differences among all factors. The Wald test was non-significant (value=0.104, df=1, p=0.7465). Together, these findings suggest that PrAS-NG22 is fully invariant regardless of gestational age.

**Table 4:** Measurement invariance of the shortened Pregnancy-related Anxiety Scale (PrAS-NG22) across gestational-age groups of pregnancy in Northern Ghana

	*χ^2^*	*df*	*CFI*	*TLI*	*RMSEA*	*ΔCFI*	*ΔTLI*	*ΔRMSEA*	
**Configural**	518.849*	346	0.932	0.910	0.058	**0.003**	**0.002**	**0.0**	1 vs 3
**Metric**	523.353*	359	0.936	0.917	0.056	**0.004**	**0.007**	**0.002**	2 vs 1
**Scalar**	553.412*	372	0.929	0.912	0.058	**0.007**	**0.005**	**0.002**	3 vs 2

Bold values indicate invariance is met. The first/second trimester: n=139; the third trimester: n=154. Study design: cross-sectional. Year: 2025. Setting: hospitals, health centers, and Community-Based Health Planning and Services (CHPS) in Northern Ghana. Sample size: 293.

* p<0.05.

### Discriminant and convergent validity

The correlation with GAD-2 (r=0.289, p<0.001) was significant but weak, supporting convergent validity. In contrast, the correlations with Kessler-6 (r= -0.217, p<0.001) and EPDS (r= -0.289, p<0.001) were significant but negative, not supporting convergent validity. The correlation with WEMWBS was non-significant (r= -0.06, p=0.193), indicating that discriminant validity was not achieved overall. However, in the first/second trimester group (r= -0.17, p<0.001) and the nulliparous group (r= -0.18, p<0.001), statistically significant weak negative correlations between the PrAS-NG22 and WEMWBS scales were found, suggesting evidence of discriminant validity within these subgroups.

### Reliability

The PrAS-NG showed excellent internal consistency (α=0.907), with the PrAS-NG22 performing even better (α=0.937). The subscales also had consistently high internal consistency, except medical/physical pain (α=0.769) and delivery concerns (α=0.581). The PrAS-NG22 also demonstrated acceptable reliability across groups (Table 5). The ICC for test-retest reliability for the PrAS-NG22 indicated consistency in the measure over time (ICC=0.808).

**Table 5:** Reliability of the shortened Pregnancy-related anxiety scale (PrAS-NG22) across subgroups of pregnant women in Northern Ghana

*Characteristics*	*Age Group^a^*		*Gestation^b^*		*Parity Status^c^*	
	≤24 years	>24 years	First and second trimester	Third trimester	Multiparous	Nulliparous
Cronbach’s alpha	0.915	0.924	0.923	0.919	0.920	0.925

a Age (≤24: n=96; >24 years: n=196).

b Gestational age groups: first and second trimester n=139; third trimester n=154.

c Parity status: nulliparous n=60, multiparous n=233.

Study design: cross-sectional. Year: 2025. Setting: hospitals, health centers, and Community-Based Health Planning and Services (CHPS) in Northern Ghana. Sample size: 293.

*p<0.05.

## DISCUSSION

In this study, we aimed to assess the psychometric validity of the 52-item adapted version of the Pregnancy-related Anxiety Scale (PrAS-NG) in three districts of Northern Ghana: Savelugu, Mion, and Tamale Metropol. We find that the PrAS-NG is a valid and reliable tool for assessing pregnancy-related anxiety in northern Ghana, exhibiting strong face, content, and construct validity. We find that the reduced length, 22-item PrAS-NG22 is a strong measure, consisting of nine culturally relevant factors, with good fit indices, convergent validity with some related constructs (psychological distress, generalized anxiety), and with strong internal reliability and test-retest reliability.

The PrAS-NG22 scale was a consistent measure of PrA regardless of women’s age, gestational age, and parity. The PrAS-NG22 also demonstrated acceptable internal reliability among all subgroups mentioned above, indicating that the items within the scale consistently measure the construct of PrA across different demographic factors in northern Ghana.

This study shed light on the differences in PrA between Ghanaian and the high-income country contexts where most of the other measures have been developed. Some concerns were consistent across contexts, but there were also many differences. Factors that were shared between Ghanaian women and those in high-income communities were worries about childbirth, concerns about taking on the responsibilities of motherhood, anxieties about medical staff, and worries about the baby’s health. However, some areas were commonly observed in HICs but were not viewed as important elements of PrA for northern Ghanaian pregnant women. These included body image concerns, acceptance of pregnancy, avoidance factors (such as preferences for the mode of delivery), and anxiety indicators like panicking without reason or not feeling content.

The pregnant women in northern Ghana expressed concerns rooted in their socio-cultural norms and values. For instance, we had expected that Ghanaian women would have similar concerns about the safety and long-term effects of cesarean sections as women in high-income countries, based on the Naa Gandau et al.^27^ study in Ghana. However, we found that northern Ghanaian women feared being stigmatized or labelled as unfaithful if they considered having a cesarean section. By contrast, women in HICs were more worried about the short-term and long-term safety of cesarean sections as a way of giving birth. As expected from the Biaggi et al.^28^ systemic review, the women in our study were concerned about childbirth expenses and the possibility of paying for maternal health services, whereas this is uncommon in high-income contexts^29^. Cultural norms, healthcare disparities, infrastructure limitations, and levels of education and awareness may explain the unique ways of perceiving and understanding PrA in Ghana^6^.

The PrAS-NG22 demonstrated convergent validity with measures of generalized anxiety and psychological distress, with higher PrA scores being associated with higher levels of general anxiety and distress. This is consistent with the original development of the PrAS in Australia^28^, which found that the PrAS was positively associated with a measure of generalized anxiety. However, we found a relationship which was opposite to what we expected between depressive symptoms and PrA, with lower depressive symptoms being associated with more PrA. This is opposite of expectations and of previous research^29,30^. Additionally, while weak negative correlations were observed between the PrAS-NG22 and the WEMWBS in some subgroups (first and second trimester women, and nulliparous women), no significant relationship was found in the overall sample, suggesting limited discriminant validity with wellbeing. Indeed, this is inconsistent with previous work, which tends to find that wellbeing is negatively associated with PrA^29-31^. These results highlight potential differences in how PrA manifests and is measured across cultural contexts. Given that there have not been validation studies of them in northern Ghana, one possibility is that the EPDS and WEMWBS may not fully capture aspects of perinatal mental health relevant to pregnant women in this region. Another possibility is that – while we used translation and back translation for these measures and had them reviewed by a local advisory committee – there may have been issues with the translated versions of these measures.

### Strengths and limitations

We conducted a thorough adaptation process and psychometric evaluation to try to develop the strongest possible measure for use with pregnant women in northern Ghana speaking either Dagbani or English. However, the study has some limitations. First, although we established face, content, construct, convergent, and discriminant validity, we were not able to evaluate the PrAS-NG22 for criterion or clinical validity. Second, we used stratified purposive sampling from antenatal clinics while women attend antenatal appointments in Ghana, and so we believe this is likely to have captured a range of participants, but we did not include any participants who for whatever reason did not attend antenatal appointments. Finally, the PrAS-NG22 showed weak or no correlation with some commonly used measures of mental health (e.g. EPDS, WEMWBS), suggesting the need for further investigation into its specificity.

## CONCLUSIONS

The validation of the PrAS-NG22 represents a significant step towards addressing the gap in culturally appropriate tools to assess PrA among women in Africa. The scale demonstrated strong psychometric properties, including reliability, construct validity, and measurement invariance across key demographic groups. By incorporating locally relevant concerns, it provides a nuanced understanding and assessment of PrA that differs from high-income country contexts. However, further research is needed to explore its predictive validity, applicability in clinical settings, and performance in longitudinal studies. Ultimately, the PrAS-NG22 has the potential to improve the identification and support of pregnant women experiencing anxiety, contributing to better maternal and neonatal health outcomes, and allowing for the evaluation of interventions to reduce PrA.

## Supplementary Material



## Data Availability

The data supporting this research are available from the authors on reasonable request.
