# Supplementary material for: Validation of the adapted pregnancy-related anxiety scale in Northern Ghana (PrAS-NG)
Source: Eur J Midwifery. 2026 Mar 20;10:10.18332/ejm/215188. doi: 10.18332/ejm/215188 (PMC13011984; doi:10.18332/ejm/215188)
Supplement: Supplementary file 1 [file EJM-10-08-s1.pdf]

Appendix A: Dagbani and English Versions of the PrAS-NG Items Used in a Cross-Sectional Study Among Pregnant Women (n = 586) in Northern Ghana (August–September 2021)

The English Items	The Dagbani Items
1) I worry that I won't be able to organize clothes for my baby before my delivery date.	Di teharimami ni nkutoi laɣisi m-bileɣu maa nema pɔin ka o sheena.
2) I worry about not getting all the necessary delivery items before my delivery date.	Di teharimami ni nkutoi laɣim doɣim bin chehi chehi pɔin ka ndoɣim saha paagi.
3) I know that the naming ceremony of my baby will be well organized.	M-mi ni nbiiŋ̍o suuna dalli binsheɣu kam ni chang venyela.
4) I worry about unnecessary interventions during delivery (e.g., forceps use during delivery).	Di teharimami zanŋ̍kpa vihigu taligu nitiyen dɔɣi (eg maɣsi yuusibu).
5) I worry that I will tear or need to be cut during child birth.	Di teharimami ni n-tooni nitoi tahi bei kabi pahi ntooni niti yen dɔɣi.
6) When I think of childbirth, I know that I will cope with the pain.	Mandei ni teegi dɔɣim yela, mmi nini ntoi deegi berim sheli dinbe di puuni.
7) I feel confident that I will be fine during childbirth.	N-dihi tabli ni binsheɣu kam ni chanŋ̍ ven-yela n dɔɣim saha.
8) I am worried about being restrained in some way and not able to move during delivery.	Di teharimami zanŋ̍kpa ndoɣim saha biyiti yeli nin donmi shem nibi bora, bei kabi yeli ni ndi voori m-maŋ̍a.
9) I fear losing control of my body during labour.	Dabɛm malimami zangkpa ndoɣim saha diyi nyɛla mbilan nsu n-ningbina.
10) I worry that the doctors/midwives will leave delivery tools (gauze, forceps etc) in me and close me up.	Di teharimami ni n-doɣim saha doctanimmaa ni tam bi doɣim binchehi kaman maɣsi n-ning npulli ni ka sheli.
11) I fear that I may have prolonged labour before delivery.	Dabɛm malimami ni nti walisi yuugi ndoɣim saha.
12) I am uncertain about the mode of delivery that I will have.	M-bimi niti yen doɣi shem, diyen nyela tooni doɣibu bei apoleshin kabi yen n-ninŋ̍ma.
13) I worry that the midwives will slap/hit my thighs while in labour.	Di teharimami ni nursi nimmaa (midwives) ni ŋ̍mema gbinpaɣasi bei ŋ̍me n-gbalipina tapaɣsi n doɣim saha.
14) I worry that if my labour sets in at night, I won't have good care.	Di teharimami ni doɣim ni kpurgima yunŋ̍ saha, nkunye lihiri sunŋ̍ ashibtini.

15) I worry that I will not be able to deliver per vagina.	Di teharimami ni nkutoi doɣi tooni doɣibu.
16) I worry that I won't do a good job as a mother.	Di teharimami ni ntoi nye ma yoli bei ma so ήun nkutoi yoli o bii venyeliga.
17) I worry that I may not have enough breast milk for my baby.	Di teharimami ni nbihi kom ku galisi nti mbia.
18) I look forward to meeting my baby.	N-kuli dzimi nguli nbii ήɔ paabu na.
19) This pregnancy is very much wanted.	Nbori n-pulli ήɔ pam.
20) I am worried that I am not getting sex from my husband because I am pregnant.	Di teharimami ni mpuliήo ncheka Nyidana bilan doondima.
21) I know the health workers will not physically or verbally abuse me.	Mmi ni doctaninmaa ni nurseninmaa ku tuma bei nήmema.
22) I know that midwives/doctors will be kind.	Mmi ni doctaninmaa ni nursenimaa bori niriba yeltoɣa.
23) I know that midwives/doctors will be helpful.	Mmi ni doctaninmaa ni nursenimaa ni sonήma.
24) I know that I can ask the midwives/doctors anything.	Mmi ni nitoi bohi doctaninmaa ni nurseninmaa binsheɣu kam.
25) I know that the health workers are competent.	Mmi ni doctaninmaa ni nursenimaa mi bi tuma venyelinga.
26) I think that caesarean birth is safer than a vaginal birth.	Mmi ni operation maa mali alaafei ngari tooni dɔɣim.
27) I worry that I may not conceive again if I have to undergo a caesarean section.	Di teharimami ni nkutoi lan kpɣi pulli mandei ni ning operation dɔɣim.
28) I worry that I will be stigmatized if I have to go through CS.	Di teharimami ni bin ganήbu mandei ni dɔɣi operation dɔɣibu.
29) I worry that people will think that I am weak if I deliver in the hospital.	Di teharimami ni niribi ni tehi ni anka yaa nninήgbinani mandei ni dɔɣi ashiptini.
30) I worry that my husband may think that I am unfaithful if I am unable to deliver per vagina.	Di teharimami ni nyidan nitoi tehari ni nyila sambani dindeeni ninή kanbitoi doɣi tooni dɔɣim.
31) I worry about what I will do if my baby is not normal.	Di teharimami niyen n-ninή shem dindeening ka n-bii maa nahim gbana bi paaigi.
32) I worry about having a sick or disabled baby.	Di teharimami ni n-bii maa nni tooi ka alaafei bei ka o mali dalinή.

33) I worry about the sex of my baby.	Di teharimami zankpa mbiima sifa (sex)-doo bei paga
34) I worry about the cost of childbirth.	Di teharimami zanήkpa dɔɣim laɣi chehi chei dibu.
35) I worry that I may have to pay out of pocket for childbirth.	Di teharimami ni ndɔɣim saha ashipti nimmaa ni cheka nyo laɣisheήa.
36) I worry that I am not able to do my daily activities due to the pregnancy.	Di teharimami ni ntoi kani dɔɣim saha.
37) I worry that I may lose my life through childbirth.	Di teharimami ni ntoi kani dɔɣim saha.
38) I worry that I will have still birth.	Di teharimami ni ni ntoi dɔɣi mbii maa ka oka nyevili.
39) I worry that I will have miscarriage.	Di teharimami ni ni ntoi zanή puliήo ndoli shinsheɣu.
40) I am excited when I feel my baby kicking.	N suhi paligiri pam mandei niwum mbii maa ni damdi npulini.
41) I worry that I will not get the best of care from health staff during my pregnancy.	Di teharimami ni doctanimmaa ni nursinimaa kutima lihiri sunή npuliήo polo.
42) I worry that the health workers will not give me the correct expected date of delivery.	Di teharimami ni ni ntoi nye dzi pooli nina gbubi pulliήo bei dɔɣim saha.
43) I worry that a pre-existing health condition will affect my pregnancy or baby.	Di teharimami ni dori sheli dinbe n-ningbina ni ni toi saɣim npulli ήɔ bei nbii maa.
44) I worry about the cost of transport to the health facility for ante-natal care or delivery.	Di teharimami zankpa laɣifu sheli ni yen di nchang zahimbu bei n-kpuɣi loori bei motor nchang zahimbu bei ni yen doɣi.
45) I worry about having to take a bad road to access health care with my pregnancy.	Di teharimami zankpa so beɣu dolibu ni yen nchang ashiptini kadi nyela npulli ήɔ zugu.
46) I worry that I won't get access to a vehicle if I am referred to a higher facility to deliver.	Di teharimami ni nku-nya loori nchang ashipti titalini ashipti bila nim ni yeli ni nchamg ashipti titalini.
47) I worry about being in labour for a long time at home.	Di teharimami zankpa nti wolisi yuu yiήa doɣim saha
48) I fear that I won't get enough support from my husband or partner during my pregnancy.	Dabem malimami ni nyidana ku sonήma venyela npulli ήo shei.
49) I fear I won't get enough support from my mother in-law during my pregnancy.	Dabem malimami ni nyidan ma ku sonήma venyela npulli ήo shei.

50) I am worried because I don't have enough money to ensure a healthy pregnancy.	Di teharimami ni npulli ŋ̗o alaafei ku chang venyla lagfu kalinsi zugu.
51) I worry that my pregnancy or baby will be attacked spiritually.	Di teharimami niso nitoi zang shiriku ndolli npulli ŋ̗o bei biso ŋ̗un be npulli ŋ̗oni.
52) I worry about taking pregnancy-related medications.	Di teharimami zankpa paɣapuu tisheŋ̗a bini yelli niti valimimaa.

Note. Likert scale responses 1 (not at all-), 2 (occasionally-), 3 (quite often-), 4 (very often-)

PrAS-NG is Pregnancy-related Anxiety Scale of Northern Ghana

Appendix B: The Confirmatory Factor Analysis Model of the 22-item Pregnancy-related Anxiety Scale of Northern Ghana (PrAS-NG22) from a Cross-Sectional Study Among Pregnant Women (n = 586) Conducted Between August and September 2021 in the Tamale Metropolis, Savelugu Municipality, and Mion District, Northern Region of Ghana

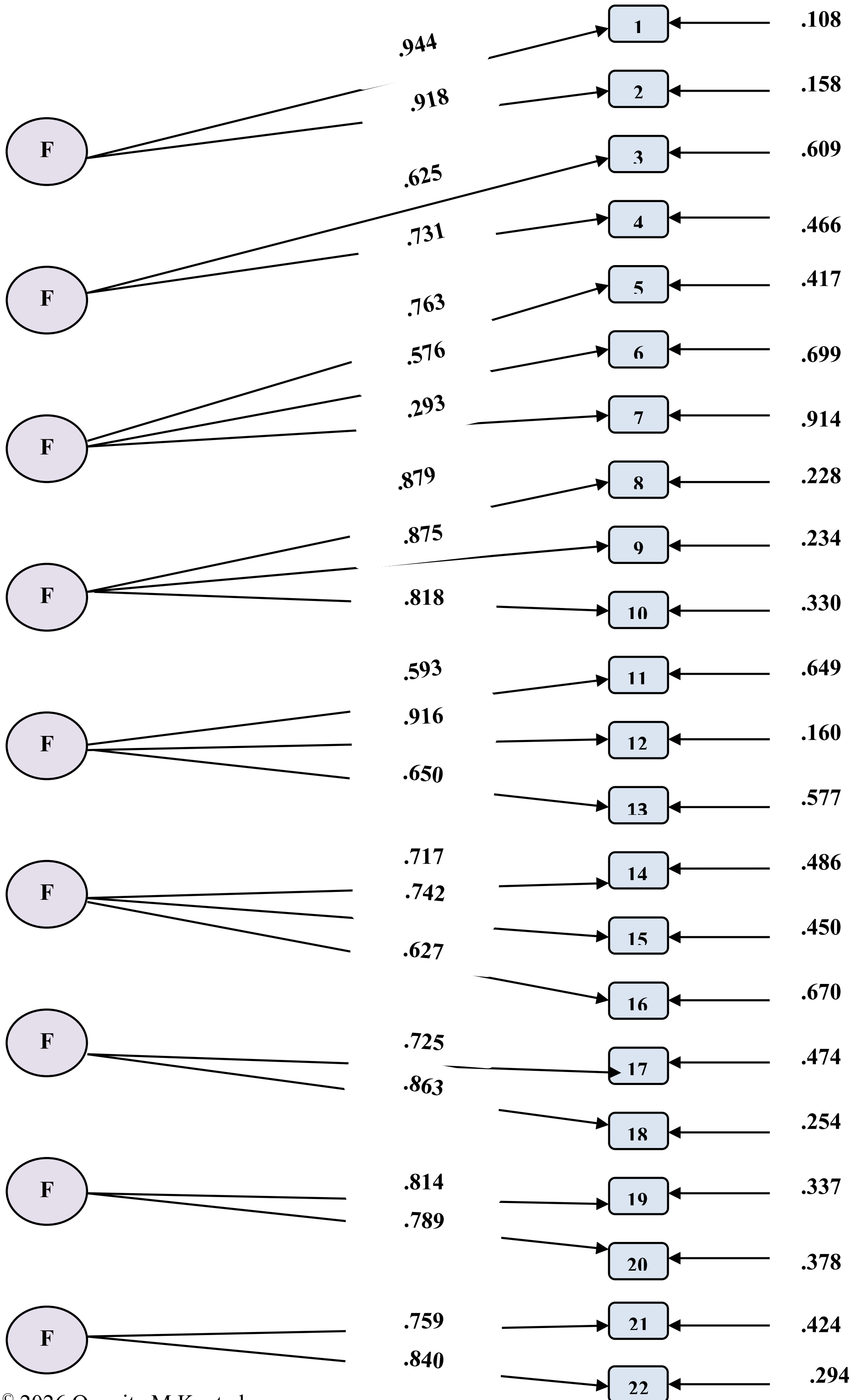

© 2026 Qannita M.K. et al.
